# MF-TopoNet: A Multi-Frequency Topological Neural Network for Epileptic Seizure Prediction

**DOI:** 10.3390/s26175623

**Published:** 2026-09-04

**Authors:** Yingchun Mei, Jialu Sun, Dawan Wang, Jianpeng An, Haoyu Li, Jiahua Li

**Affiliations:** 1 School of Electrical and Information Engineering, Tianjin University, Tianjin 300072, China; meiyc@chec.com.cn (Y.M.); sunjialu_7@connect.hku.hk (J.S.); dawan123@tju.edu.cn (D.W.); anjianpeng@tju.edu.cn (J.A.); 2 Huadian Heavy Industries Co., Ltd., Beijing 100070, China; 3 School of Human Settlements and Civil Engineering, Xi’an Jiaotong University, Xi’an 710049, China; 4 Beijing Everloyal Technology Co., Ltd., Beijing 100085, China

**Keywords:** seizure prediction, electroencephalogram, brain network topology, convolutional neural network

## Abstract

Electroencephalogram (EEG)-based seizure prediction has recently emerged as a critical technique for clinical diagnosis and intervention. However, conventional multi-channel EEG analysis methods often overlook the brain’s intrinsic spatial topology and typically employ fixed channel ordering, which constrain their ability to capture cross-regional interactions effectively. To address these limitations, this study proposes a novel Multi-Frequency Topological Neural Network (MF-TopoNet) that jointly captures topological and spatial–temporal characteristics of EEG signals. The proposed framework leverages both constructed functional brain networks and raw multi-channel EEG recordings as inputs, thereby facilitating complementary feature extraction. Specifically, the TopoConv module integrates topological information into the convolutional process and adopts randomized channel fusion to enhance feature diversity. In addition, a cross-band attention mechanism is introduced to model interactions across multiple frequency bands, further improving prediction accuracy. Extensive experiments conducted on the CHB-MIT and Siena datasets demonstrate the superiority and robustness of MF-TopoNet. Under 10-fold cross-validation, the proposed model achieved 95.88% accuracy, 95.60% sensitivity, and 96.15% specificity on the CHB-MIT dataset and 94.01% accuracy, 93.92% sensitivity, and 94.11% specificity on the Siena dataset. These results underscore the importance of incorporating brain topology into deep learning frameworks and highlight the effectiveness of multi-frequency feature fusion for improving seizure prediction performance.

## 1. Introduction

Epilepsy is a chronic brain disorder characterized by irregular interruptions of normal brain function, affecting approximately 65 million people worldwide [1]. The symptoms of epilepsy manifest through signs such as cramps, loss of concentration, convulsions, and involuntary movements, which often occur unpredictably and pose significant risks to patients, particularly during activities such as driving or swimming. While epilepsy seizures can be managed using medication, challenges still remain for regions with limited medical resources and individuals with drug-resistant epilepsy [2]. Consequently, the development of accurate and timely seizure prediction methods holds immense clinical value, offering the potential to alleviate patients’ concerns about irregular seizures and significantly enhance their quality of life.

Electroencephalography (EEG), a widely used technique for recording the brain’s electrical signals, plays a crucial role in diagnosing epilepsy [3]. In recent years, multi-channel EEG has gained significant attention in the field of epilepsy seizure prediction due to its advantages, including non-invasiveness, ease of acquisition, and high temporal resolution [4]. By continuously capturing electrical brain activity, multi-channel EEG enables the classification of epileptic signals into three distinct phases: interictal (a state of normal brain activity between seizures), preictal (the transitional period preceding a seizure), and ictal (the seizure episode itself). This categorization provides a foundational framework for modeling seizure prediction as a binary classification task, which aims to distinguish preictal states from interictal ones.

The brain’s spatially distributed architecture, partitioned into functionally distinct regions, poses unique challenges for EEG analysis. While multi-channel EEG signals inherently capture spatial patterns from various brain regions, current methodologies often fall short in effectively leveraging their long-range functional dependencies. To bridge this gap, innovative approaches are required to dynamically integrate both local and global inter-channel interactions, a critical yet unresolved challenge in EEG-based seizure prediction. With the advancement of deep learning, numerous frameworks have been proposed to process multi-channel EEG signals by reducing noise and extracting deeper features. Among these, convolutional neural networks (CNNs) have shown practical utility. For instance, Gao et al. [5] designed a dual-scale CNN with multiple kernel sizes to capture morphological features at different spatial resolutions. Hussein et al. [6] converted EEG signals into images and employed a semi-dilated convolution module to expand the receptive field along the image width while preserving spatial resolution along the height. Despite recent progress, CNN-based approaches remain constrained by their reliance on traditional convolutional layers, which limit flexibility in channel fusion. These layers typically use fixed kernel sizes along the spatial (channel) dimension and impose a local receptive field, restricting each channel’s response to a small neighborhood of adjacent channels. For instance, the activation at channel *i* depends only on nearby channels and lacks access to distant, non-adjacent ones. This locality bias impairs the modeling of long-range dependencies across spatially distributed EEG channels and hinders comprehensive cross-channel integration. To overcome this, more adaptive fusion mechanisms are needed to capture global interactions beyond local neighborhoods.

Furthermore, as an intuitive representation of brain states, multi-channel EEG signals not only exhibit complicated fluctuation characteristics like nonlinear time series, but also possess certain complex system properties [7]. EEG-derived brain networks encapsulate the intricate connectivity patterns and interactions among different brain electrodes, intrinsically representing the topological features of the brain. Epileptic seizures disrupt the topology of functional brain networks by inducing hyper-synchronized neuronal activity. These disruptions lead to dynamic alterations in connectivity patterns, making topological metrics relevant for characterizing epileptic states. Recent years have witnessed a growing body of research exploring brain networks and their topological properties to identify abnormal neural activity. For example, Xu et al. [8] used dynamic functional connectivity to construct brain graphs and extracted non-Euclidean features from multi-channel EEG signals. Gao et al. [9] introduced a stepwise multivariate Granger causality model to represent the hierarchical and directional structure of brain networks. Peng et al. [10] calculated intra- and inter-hemispheric connectivity to analyze network organization in the epileptogenic zone. However, current deep learning methods often overlook the inherent topological information in the brain. This oversight has impeded the ability of deep learning models to accurately decode the intricate dynamics underlying epileptic seizures.

Additionally, the brain exhibits clear frequency-dependent properties. In patients with epilepsy, EEG signals across different frequency bands are closely associated with seizure activity. So far, many studies have attempted to explore the obscure patterns of epileptic seizures hidden in EEG signals from different frequency bands. For example, Yan et al. [11] extracted sub-band features using wavelet decomposition and applied feature selection based on the maximal relevance and minimal redundancy criterion for seizure prediction. Anuragi et al. [12] decomposed EEG signals into sub-bands via empirical wavelet transform and independently analyzed each band for epileptic state classification. It is found that these methods primarily focus on frequency-specific information, yet do not explicitly model interactions across different frequency bands. From a neuroscience perspective, the brain operates as an integrated system in which frequency bands interact dynamically. Each band influences and is influenced by others, and no frequency component functions in isolation. Capturing these cross-band interactions is essential for accurately modeling brain dynamics. Therefore, developing methods that can comprehensively characterize inter-frequency dependencies and leverage their complementary properties is crucial for improving EEG-based seizure prediction.

Inspired by the background discussed above, we propose a novel deep learning framework for epilepsy prediction that integrates the topological features of functional brain networks into the convolutional process, enabling flexible and dynamic channel fusion, and simultaneously fuses topological information across multiple frequency bands. The framework is driven by three key innovations that contribute to its superior performance:

1. A Multi-Frequency Topological Neural Network (MF-TopoNet) is proposed for epileptic seizure prediction, capable of capturing the spatio-temporal dynamics of brain activity and improving prediction accuracy within individual subjects.
2. A novel convolutional mechanism is designed to integrate brain network topological features into the convolutional process, addressing the limitations of traditional convolutional layers by enabling more flexible and adaptive inter-channel feature fusion.
3. A multi-frequency fusion strategy based on a multi-head attention mechanism is developed, enabling the integration of complementary topological information from different frequency bands to enhance the robustness of seizure prediction.

## 2. Methodology

**Figure 1:**
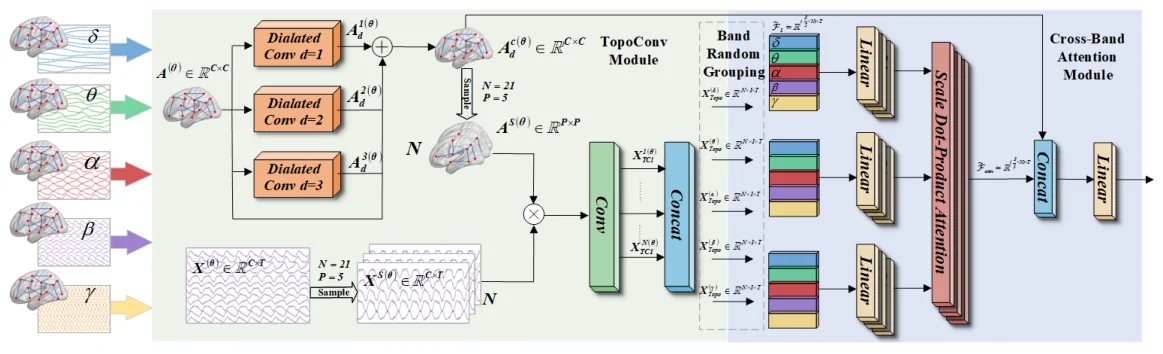
The architecture of the proposed MF-TopoNet model.

This section provides a detailed introduction to the design concept and model architecture of MF-TopoNet. As shown in Figure 1, MF-TopoNet takes multi-channel EEG signals from five frequency bands (δ: 0.5–4 Hz, θ: 4–8 Hz, α: 8–12 Hz, β: 12–30 Hz, and γ: 30–50 Hz) associated with epileptic seizure as input. For each frequency band, the corresponding EEG signal is denoted as $X^{\left(f\right)}\in R^{C\times T}$, where *C* is the number of channels, *T* is the number of time points, and f∈{δ,θ,α,β,γ}. To model the underlying topological structure of brain activity relevant to epileptic seizure, an individual brain network G(V,E) is constructed for each frequency band. The network is represented by an adjacency matrix $A=\left(a_{ij}\right)\in R^{C\times C}$, where each node $v_{i}\in V$ corresponds to an EEG channel, and each edge weight $a_{ij}$ denotes the connection strength between nodes $v_{i}$ and $v_{j}$. Edges are determined based on the normalized mutual information (MI) between EEG signals recorded at different electrodes, reflecting the amount of shared information. To retain the most relevant connections, only the top 25% of edges with the highest MI values are preserved for further analysis.

Subsequently, a TopoConv module is proposed to process brain topology and EEG signals at a single frequency band. Taking the θ band as an example, the module receives EEG signals $X^{\left(\theta \right)}\in R^{C\times T}$ along with the corresponding brain network, represented by the adjacency matrix $A^{\left(\theta \right)}=\left(a_{ij}\right)\in R^{C\times C}$. To capture diverse topological patterns associated with brain dynamics, the module first applies a dilated convolutional group to the input matrix $A^{\left(\theta \right)}$, followed by a ReLU activation and batch normalization (BN). This group comprises three dilated convolutional layers with different dilation rates, allowing the model to capture multi-scale topological structures encoded in functional brain connectivity.

(1) $$\left\{\begin{array}{c} A_{d}^{1(\theta )}={\mathrm{D-Conv}}_{1}\left(\omega ,A^{\left(\theta \right)},d_{1}\right)+b\\ A_{d}^{2(\theta )}={\mathrm{D-Conv}}_{2}\left(\omega ,A^{\left(\theta \right)},d_{2}\right)+b\\ A_{d}^{3(\theta )}={\mathrm{D-Conv}}_{3}\left(\omega ,A^{\left(\theta \right)},d_{3}\right)+b, \end{array}\right.$$

where D-Conv(·) denotes a dilated convolution operation, and ω and *b* are the shared weight matrix and bias term. The input to all three layers is the adjacency matrix $A^{\left(\theta \right)}$, and the convolution kernels are 3×3 in size. The dilation rates $d_{1}$, $d_{2}$, and $d_{3}$ are set to 1, 2, and 3, respectively. To enhance the multiscale representation of brain network topology, features extracted from different receptive fields are aggregated through an additive combination:

(2) $$A^{c(\theta )}=A^{\left(\theta \right)}+A_{d}^{1(\theta )}+A_{d}^{2(\theta )}+A_{d}^{3(\theta )},\;A^{c(\theta )}\in R^{C\times C}.$$

Next, to enable more flexible and dynamic fusion of brain information across channels, a novel topological convolutional (TopoConv) layer is introduced. Specifically, random cross-channel sampling is employed to encourage non-local interactions across the EEG channel space, especially between distant channels, and reduce over-reliance on predefined spatial neighborhoods. Given the θ-band EEG signal $X^{\left(\theta \right)}\;\in \;R^{C\times T}$ and its topology-enhanced adjacency matrix $A^{c(\theta )}\;\in \;R^{C\times C}$, the sampling procedure selects *p* indices to generate a sub-signal $X^{S(\theta )}\;\in \;R^{p\times T}$ and a corresponding sub-network $A^{S(\theta )}\;\in \;R^{p\times p}$. Specifically, *p* is a fixed hyperparameter rather than a learnable parameter. In this work, we set p=5. A topological interaction operator is then applied to enhance feature representation:

(3) $$X_{\mathrm{TC}}^{\left(\theta \right)}=\mathrm{Conv}\left(\omega ,\;T\left(A^{S(\theta )},X^{S(\theta )}\right)\right)+b,\;X_{\mathrm{TC}}^{\left(\theta \right)}\in R^{D_{1}\times 1\times T},$$

where $T(A^{S(\theta )},X^{S(\theta )})$ denotes the topological interaction operator (e.g., vector product), and $D_{1}$ is the number of convolution kernels. After pooling and concatenating $X_{\mathrm{TC}}^{\left(\theta \right)}$ with $A^{S(\theta )}$, the resulting feature matrix $X_{\mathrm{TC}1}^{\left(\theta \right)}$ is obtained. The TopoConv layer is repeated *N* times, and the outputs are concatenated to produce the final topology-enhanced representation:

(4) $$\begin{array}{cc} X_{1}^{\left(\theta \right)} & =\mathrm{Concat}\left(X_{\mathrm{TC}1}^{1(\theta )},X_{\mathrm{TC}1}^{2(\theta )},\ldots ,X_{\mathrm{TC}1}^{N(\theta )}\right),\\ X_{1}^{\left(\theta \right)} & \in R^{N\times 1\times T}, \end{array}$$

where N=21 in this work. This random permutation is applied to the learned topology-aware feature maps rather than the original EEG electrode order. Therefore, the functional spatial relationships encoded by the adjacency matrix are preserved during TopoConv. The permutation mainly serves as a stochastic grouping strategy to reduce fixed grouping bias and improve feature diversity in cross-band fusion.

Further feature extraction is applied to $X_{1}^{\left(\theta \right)}$ using a convolutional layer, resulting in $X_{2}^{\left(\theta \right)}$. The final output is obtained by element-wise summation:

(5) $$X_{\mathrm{Topo}}^{\left(\theta \right)}=X_{1}^{\left(\theta \right)}+X_{2}^{\left(\theta \right)},X_{\mathrm{Topo}}^{\left(\theta \right)}\in R^{N\times 1\times T}.$$

For the five frequency bands, the resulting topology-aware features are denoted as $\left\{X_{\mathrm{Topo}}^{\left(\delta \right)},X_{\mathrm{Topo}}^{\left(\theta \right)},X_{\mathrm{Topo}}^{\left(\alpha \right)},X_{\mathrm{Topo}}^{\left(\beta \right)},X_{\mathrm{Topo}}^{\left(\gamma \right)}\right\}$, which are then passed to the cross-band attention module to capture interdependencies among spectral components. Specifically, to facilitate more effective cross-band interaction, each frequency-specific representation is first processed by randomly permuting its feature channels to prevent fixed spatial bias. The permuted channels are then divided into three equally sized groups as follows:

(6) $$\left\{\begin{array}{c} G_{1}^{f}={\left\{\pi_{f}\left(k\right)\right\}}_{k=1}^{N/3},\\ G_{2}^{f}={\left\{\pi_{f}\left(k\right)\right\}}_{k=N/3+1}^{2N/3},\\ G_{3}^{f}={\left\{\pi_{f}\left(k\right)\right\}}_{k=2N/3+1}^{N}, \end{array}\right.$$

where each $\pi_{i}$ denotes a random permutation of the *N* feature maps in the *f*-th frequency band, thereby encouraging stochastic channel grouping. The grouped features are then integrated across frequency bands by aligning groups with the same index m∈{1,2,3}, and concatenating them as:

(7) $$\begin{array}{cc} {\tilde{F}}_{m} & =\underset{f\in \{\delta ,\theta ,\alpha ,\beta ,\gamma \}}{\mathrm{Concat}}\left(X_{\mathrm{Topo}}^{\left(f\right)}\left[:,G_{m}^{f},:\right]\right),\\ {\tilde{F}}_{m} & \in R^{\left(\frac{5N}{3}\right)\times T},\;m\in \left\{1,2,3\right\}. \end{array}$$

To model dynamic correlations between frequency bands and enhance inter-band interaction, a hierarchical attention framework is employed to process the group-aligned features:

(8) $$\begin{array}{cc} \mathrm{MHA}(Q,K,V) & =\mathrm{Concat}\left({\mathrm{head}}_{1},\ldots ,{\mathrm{head}}_{h}\right)W^{O},\\ {\mathrm{head}}_{i} & =\mathrm{softmax}\left(\frac{\left(QW_{i}^{Q}\right){\left(KW_{i}^{K}\right)}^{⊤}}{\sqrt{d_{k}}}\right)VW_{i}^{V},\\ F_{\mathrm{attn}} & =\mathrm{MHA}\left({\tilde{F}}_{1},{\tilde{F}}_{2},{\tilde{F}}_{3}\right),\;F_{\mathrm{attn}}\in R^{(5N/3)\times T}. \end{array}$$

where $\left\{W_{i}^{Q},W_{i}^{K},W_{i}^{V}\right\}$ denote the learnable projection matrices for the query, key, and value in the *i*-th attention head, and $W^{O}$ is the output projection matrix. This mechanism enables the model to capture diverse inter-band dependencies, with each attention head attending to distinct spectral interaction patterns.

Finally, the obtained representation $F_{\mathrm{attn}}$ is subsequently flattened along the channel and time dimensions and concatenated with the flattened topological features derived from each frequency-specific adjacency matrix $A^{c(f)}$. The resulting high-level feature vector is then fed into fully connected layers for seizure prediction.

## 3. Experiments

### 3.1. Datasets

In this work, we employed two publicly available datasets, CHB-MIT Scalp EEG and Siena scalp EEG, which are widely used in epileptic seizure prediction research.

#### 3.1.1. CHB-MIT Dataset

The CHB-MIT Scalp EEG dataset was provided by Boston Children’s Hospital and the Massachusetts Institute of Technology. It consists of long-term scalp EEG recordings from pediatric patients with refractory epilepsy. EEG signals were recorded at a sampling rate of 256 Hz, with electrode placements conforming to the international 10–20 system. Each session utilized 23 channels; however, one redundant channel was excluded, leaving 22 channels for analysis. To maintain dataset balance, we selected data from 16 patients, each with at least two documented seizures and a minimum of four seizure-free hours preceding each seizure. This resulted in a total of 68 seizures available for model training and validation.

#### 3.1.2. Siena Dataset

The Siena scalp EEG dataset was collected by the Unit of Neurology and Neurophysiology at the University of Siena. This dataset consists of video-EEG recordings from 14 adult patients with epilepsy, including 9 males and 5 females aged between 20 and 71 years. EEG signals were acquired using electrodes placed according to the international 10–20 system, with a sampling frequency of 512 Hz. Each recording contains signals from 29 EEG channels, and most sessions also provide one or two electrocardiogram (EKG) channels. In this work, recordings from 13 patients were used for experimental evaluation. Following the channel selection strategy adopted in previous studies, 10 electrodes around the central scalp region, including F9, F10, Fc1, Fc2, Fc5, Fc6, Cp1, Cp2, Cp5, and Cp6, were removed. As a result, 19 EEG channels were retained as valid inputs for model training and testing.

EEG data of two datasets were segmented into 50% overlapping 2-s windows for further analysis. For seizure prediction, the preictal window was defined as the 5–35 min preceding seizure onset, while the interictal period, representing baseline brain activity, was defined as a minimum of four hours before or after seizure offset. To minimize overfitting, interictal data segments were selected to match the duration of the preictal windows.

### 3.2. Experiment Settings

The proposed MF-TopoNet model was implemented using PyTorch 2.12.1 and executed on an Intel Xeon Silver 4210R CPU coupled with an NVIDIA A100 80GB GPU. For each patient, a subject-specific 10-fold cross-validation strategy was adopted for model training and evaluation. In each fold, the data were split into training (80%), validation (10%), and testing (10%) sets. Training parameters were set as follows: 100 epochs with a batch size of 512. The cross-entropy loss function was employed, and the Adam optimizer was used with a learning rate of 0.001.

### 3.3. Results and Analysis

**Figure 2:**
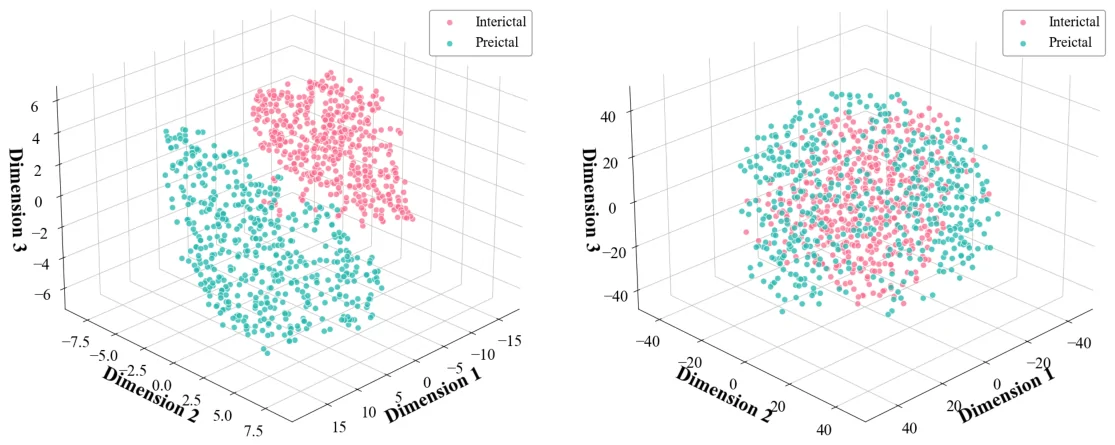
Three-dimensional t-SNE visualization of interictal and preictal states for subject chb21 from the CHB-MIT dataset using model-derived features (**left**) and raw EEG data (**right**).

Experimental evaluation on the CHB-MIT dataset demonstrates the robust performance of the MF-TopoNet model in seizure prediction. As shown in Table 1, the model achieved an average accuracy of 95.88% under 10-fold cross-validation, with a sensitivity of 95.60% and a specificity of 96.15%. As shown in Table 2, the model achieved an average accuracy of 94.01%, sensitivity of 93.92%, and specificity of 94.11% on the Siena dataset. These metrics highlight the model’s ability to generalize well across different subsets of the dataset, ensuring consistent and reliable performance in various scenarios. Further supporting this, the 3D t-SNE visualization of the model-derived features for subject chb21 (Figure 2) reveals a clear separation between interictal and preictal states in the feature space. This distinct clustering underscores the effectiveness of MF-TopoNet in capturing meaningful patterns and topological structures essential for seizure prediction. In conclusion, the MF-TopoNet model enhances seizure prediction performance by integrating topological information into feature extraction and dynamically fusing multi-frequency features based on the brain’s collaborative mechanisms.

### 3.4. Comparison Studies

We first compared the proposed MF-TopoNet with several representative deep learning models for EEG decoding and seizure prediction. To ensure a fair comparison, all baseline models were re-implemented and evaluated within the same experimental framework as MF-TopoNet. Specifically, the same subject selection, EEG preprocessing, frequency-band decomposition, 2-s window segmentation, class-balancing strategy, and subject-specific 10-fold cross-validation protocol were applied to all models. The principal architectural configurations of the baseline models followed their corresponding publications, while the data partitioning and evaluation procedures were kept consistent across all methods. Therefore, the results reported in this section were obtained from our implementations rather than directly quoted from the original publications. A brief overview of these comparison models is provided below:

CNN-BiLSTM [13]: A stacked CNN-BiLSTM framework that automatically extracts spatio-temporal features from raw epileptic EEG signals.

LightSeizureNet [14]: A lightweight seizure prediction model that employs 1-D dilated convolutions and structured pruning to remove redundant information.

EEGNet [15]: A compact convolutional neural network widely used for EEG decoding and feature extraction tasks.

EEG-Conformer [16]: A hybrid convolutional-transformer framework for EEG decoding that captures temporal dependencies through self-attention mechanisms.

STS-HGCN-AL [17]: A hierarchical graph convolutional network that integrates spatio-temporal-spectral features and active learning for patient-specific seizure prediction.

The comparative results on the CHB-MIT dataset are summarized in Table 3. MF-TopoNet achieves the highest accuracy (95.88%) and competitive sensitivity (95.60%) and specificity (96.15%), outperforming existing models in overall balance. Although its sensitivity is slightly lower than STS-HGCN-AL (97.56%) and specificity is slightly lower than EEG-Conformer (96.67%), MF-TopoNet does not suffer from any obvious weakness, making it the most robust model for seizure prediction. The improvement of seizure prediction performance is attributed to the fact that the model forms a rich spatial feature representation by adopting a dynamic channel fusion strategy in the feature extraction of multi-channel EEG signals.

### 3.5. Ablation Studies

To further investigate the effectiveness of different components in the proposed framework, ablation experiments were conducted on the CHB-MIT dataset. As shown in Table 4, the ablation studies systematically evaluate the contributions of key components in the MF-TopoNet model. Specifically:

Model 1, which removes the topology convolution module, demonstrates a significant drop in performance (accuracy: 69.71%, sensitivity: 67.49%, specificity: 73.06%). This substantial degradation underscores the critical role of the topological convolution module in capturing spatial dependencies and brain network properties.

Model 2, which replaces the dynamic multi-channel fusion strategy with 2D convolution while maintaining the same number of input channels as the EEG signal, achieves moderate performance (accuracy: 86.71%, sensitivity: 90.62%, specificity: 83.15%). However, this performance is notably inferior to that of the original MF-TopoNet, suggesting that the flexible channel fusion strategy is vital for effectively integrating multi-channel EEG information.

Compared to Model 2, our MF-TopoNet significantly reduces the number of parameters (from 4.17 M to 1.12 M), achieving a remarkable parameter efficiency while maintaining superior performance. This demonstrates that our model not only excels in computational efficiency but also effectively captures the essential features of EEG signals through its dynamic multi-channel fusion strategy.

Model 3, which removes the frequency fusion module, exhibits performance declines across all metrics (accuracy: 94.89%, sensitivity: 95.82%, specificity: 93.78%). This highlights the importance of frequency band integration in leveraging complementary information across different frequency ranges, further confirming the necessity of the multi-frequency fusion mechanism.

These ablation experiments collectively demonstrate that each component of the MF-TopoNet contributes uniquely and significantly to improving seizure prediction performance. The most substantial gains are observed when the topology convolution module, dynamic channel fusion strategy, and frequency fusion module are combined, validating the effectiveness of the model’s architectural design.

## 4. Discussion

Compared with conventional CNN-based channel fusion methods, MF-TopoNet explicitly incorporates functional connectivity into the feature extraction process. This design allows the model to exploit not only local temporal patterns from multi-channel EEG signals, but also the underlying topological relationships among EEG channels. As a result, the learned representations become more sensitive to the spatial organization and connectivity changes associated with preictal states. In addition, the cross-frequency attention mechanism further enhances the interaction among different EEG frequency bands, enabling the model to integrate complementary spectral information more effectively.

The experimental results demonstrate that the proposed MF-TopoNet can effectively distinguish preictal and interictal EEG states by jointly modeling multi-band spectral information, channel-wise interactions, and topological characteristics. On the Siena dataset, the model achieved an accuracy of 94.01%, a sensitivity of 93.92%, and a specificity of 94.11%. On the CHB-MIT dataset, MF-TopoNet achieved an accuracy of 95.88%, a sensitivity of 95.60%, and a specificity of 96.15%. Under 10-fold cross-validation, our model outperforms several representative epilepsy prediction and EEG decoding models. These results indicate that topology-guided feature extraction is beneficial for capturing discriminative EEG patterns related to seizure prediction.

## 5. Conclusions

In this paper, we proposed MF-TopoNet, a novel deep learning model for epileptic seizure prediction. The proposed framework adopts a flexible channel fusion strategy, integrates topological information into EEG feature extraction, and introduces a cross-frequency attention mechanism to model interactions among different frequency bands. By jointly extracting and integrating spatio-temporal, spectral, and topological features from multi-channel EEG signals, MF-TopoNet achieved promising performance on the CHB-MIT and Siena datasets. These results suggest that incorporating functional connectivity into deep EEG representation learning is effective for improving preictal/interictal discrimination.

Nevertheless, this study has several limitations. The experiments were conducted on CHB-MIT and Siena datasets under a subject-specific window-level evaluation protocol, which may limit generalizability. Moreover, the present study does not report false prediction rate per hour or external dataset validation, which are important for clinical deployment.

In the future, we will continue to explore the impact of effective frequency band fusion mechanisms on EEG decoding, epilepsy prediction, and leave-one-subject-out evaluation, aiming to achieve more efficient frequency band interactions.

## Figures and Tables

**Table 1:** Experimental Results for Each Patient in 10-fold Cross Validation on the CHB-MIT Dataset.

Subject	Accuracy (%)	Sensitivity (%)	Specificity (%)
01	99.74±0.35	99.68±0.52	99.80±0.24
02	97.81±0.65	97.11±0.96	98.50±1.03
03	98.24±0.55	97.54±0.90	98.94±0.60
04	97.53±0.46	97.93±0.54	97.13±1.11
05	95.84±1.04	95.14±1.12	96.54±1.21
06	87.56±1.62	88.03±1.87	87.08±2.29
07	98.67±0.80	98.81±0.74	98.52±1.15
09	95.17±0.88	96.14±0.91	94.19±1.53
10	97.75±0.34	97.79±0.62	97.71±0.54
11	94.31±1.90	93.36±3.62	95.26±1.98
16	87.44±1.32	82.36±1.51	92.52±2.51
18	96.81±0.61	96.13±0.74	97.49±0.69
20	98.78±0.28	98.40±0.46	99.17±0.59
21	99.41±0.26	99.55±0.29	99.27±0.40
22	90.62±1.26	92.81±1.73	88.42±2.40
23	98.40±0.52	98.87±0.45	97.92±0.78
Mean	95.88	95.60	96.15

Note: Values for each subject are reported as mean ± standard deviation.

**Table 2:** Experimental Results for Each Patient in 10-fold Cross Validation on the Siena Dataset.

Subject	Accuracy (%)	Sensitivity (%)	Specificity (%)
PN01	89.36±0.92	87.55±3.85	91.14±2.66
PN03	99.97±0.06	100.00±0.00	99.95±0.12
PN05	99.03±0.40	99.29±0.57	98.77±0.60
PN06	95.67±0.94	96.12±1.87	95.18±1.72
PN07	99.94±0.13	100.00±0.00	99.89±0.25
PN09	87.32±1.46	86.19±1.85	88.38±1.47
PN10	95.64±0.56	95.91±1.04	95.34±1.32
PN11	98.56±0.36	98.77±0.68	98.31±1.23
PN12	97.11±0.72	98.14±2.03	96.22±1.37
PN13	92.89±1.06	92.96±1.07	92.83±1.65
PN14	89.44±1.22	91.31±2.37	87.82±2.85
PN16	88.95±1.71	86.97±2.22	90.75±5.31
PN17	88.31±0.57	87.78±0.54	88.85±0.86
Mean	94.01	93.92	94.11

Note: Values for each subject are reported as mean ± standard deviation.

**Table 3:** Performance Comparison of Different Models on the CHB-MIT Dataset.

Model	Accuracy	Sensitivity	Specificity
CNN-BiLSTM **	94.76%	94.35%	95.17%
LightSeizureNet *	93.15%	93.19%	93.13%
EEG-Conformer *	95.16%	93.29%	**96.67%**
EEGNet **	92.13%	92.46%	91.89%
STS-HGCN-AL *	95.38%	**97.56%**	93.46%
**MF-TopoNet**	**95.88%**	95.60%	96.15%

Note: Statistical significance is reported by comparing each baseline model with MF-TopoNet. The symbols * and ** denote p<0.05 and p<0.01, respectively. The statistical test is used as supplementary evidence for the observed performance differences. The best performance in each column is shown in bold, and the second-best performance is underlined.

**Table 4:** Performance Metrics of Selected Models on the CHB-MIT Dataset.

Model	Accuracy	Sensitivity	Specificity
Model 1	69.71%	67.49%	73.06%
Model 2	86.71%	90.62%	83.15%
Model 3	94.89%	95.82%	93.78%
MF-TopoNet	95.88%	95.60%	96.15%

## Data Availability

The data used in this study are available from online open-access resources. Specifically, the CHB-MIT dataset is available at https://physionet.org/content/chbmit/1.0.0/ (accessed on 4 May 2025), and the Siena dataset is available at https://physionet.org/content/siena-scalp-eeg/1.0.0/ (accessed on 4 May 2025).
